# Epigenetic and ncRNA Regulation of CBX2 in Liver Hepatocellular Carcinoma: A Comprehensive Multi-Omics Analysis for Understanding Biological Significance

**DOI:** 10.3390/medicina62050842

**Published:** 2026-04-28

**Authors:** Kyu-Shik Lee, Jong-Heon Kim, Jongwan Kim

**Affiliations:** 1 Department of Pharmacology, College of Medicine, Dongguk University, Gyeongju 38066, Republic of Korea; there1@dongguk.ac.kr; 2 Brain Science & Engineering Institute, Kyungpook National University, 80 Daehak-ro, Buk-gu, Daegu 41566, Republic of Korea; jongheonkim@knu.ac.kr; 3 Department of Anatomy, College of Medicine, Dongguk University, Gyeongju 38066, Republic of Korea

**Keywords:** LIHC, CBX2, DNA methylation, ncRNAs, prognostic biomarker

## Abstract

*Background and Objectives:* Chromobox protein homolog 2 (CBX2), a member of the polycomb group of proteins that plays a role in chromatin remodeling, has been associated with multiple types of cancer. However, its characterization in liver hepatocellular carcinoma (LIHC) has not been fully elucidated. This study aims to systemically evaluate the expression, prognostic value, epigenetic regulation, and ncRNAs of CBX2 in LIHC. *Materials and Methods:* We performed a comprehensive in silico analysis to assess CBX2 expression at mRNA and protein levels, correlate its expression with clinical characteristics and prognosis, and explore DNA methylation and ncRNA-mediated regulatory networks. Multiple public databases (TIMER2.0, UALCAN, Human Protein Atlas, KM Plotter, MethSurv, miRNet, and ENCORI) were utilized to conduct expression, survival analysis, and construct a network encompassing miRNAs, lncRNAs, and pseudogenes. *Results:* CBX2 expression was found to be elevated in LIHC at both the mRNA and protein expression level. Increased CBX2 expression was closely linked with unfavorable clinical outcome. Methylation analysis indicated that various CpG sites, exhibiting both hypermethylation and hypomethylation patterns, were correlated with CBX2 expression and patient prognosis. Among the identified ncRNAs, hsa-miR-101-3p tended to be downregulated, whereas hsa-miR-222-3p was significantly upregulated in LIHC, and both were associated with CBX2 expression and clinical outcomes. The constructed ncRNA interaction network suggested potential associations among miRNA, lncRNAs, and pseudogenes that may be linked to tumor progression. *Conclusions:* Our results suggest that CBX2 was overexpressed in LIHC and may be associated with epigenetic alterations and ncRNA-related regulatory interactions. Its expression shows a relationship with clinical prognosis, suggesting that CBX2 could serve as a candidate biomarker. The proposed CBX2-associated ncRNA network represents a potential framework for further investigation, although additional experimental validation is required to confirm its biological and clinical relevance. Consequently, our findings suggest that CBX2 may serve as a potential prognostic biomarker and therapeutic target in LIHC, potentially influenced by specific epigenetic and post-transcriptional mechanisms.

## 1. Introduction

Liver hepatocellular carcinoma (LIHC) is the predominant form of primary liver malignancy, ranking sixth in incidence and third in cancer-associated fatalities worldwide [1]. Although diagnostic and interventional approaches have evolved, the prognosis remains poor, with a 5-year overall survival (OS) rate of approximately 18% [2]. Therefore, uncovering molecular signatures that drive LIHC initiation and progression is essential for advancing early diagnosis and tailoring individualized treatment strategies.

Chromobox protein homolog 2 (CBX2) is a member of the polycomb group family of epigenetic regulators, which mediate transcriptional repression through chromatin remodeling. Although CBX2 has been documented in breast, ovarian, and prostate cancers, its role in LIHC remains poorly defined. The mechanisms by which CBX2 expression is regulated in LIHC, and the DNA methylation and non-coding RNA (ncRNA) networks through which it may exert oncogenic functions, remain largely unexplored.

In recent decades, there has been a significant increase in interest regarding the epigenetic underpinnings of cancer and their potential applications in clinical settings. Epigenetics pertains to heritable changes in gene expression that occur without alteration in the underlying DNA sequence [3]. These regulatory mechanisms encompass DNA methylation, histone post-translational modifications, chromatin remodeling, and RNA-mediated regulatory pathways [4]. Dysregulation of these processes may contribute to aberrant gene expression patterns, including inappropriate activation of oncogenes and suppression of tumor suppressor genes, resulting in unregulated cellular proliferation, sustained inflammatory signaling, and fibrotic tissue remodeling. Over time, such alterations can facilitate local invasion and distant metastasis [5]. A considerable amount of evidence now associates epigenetic disruptions with both the initiation and progression of cancers [6,7], leading to the perspective that cancer may not solely arise from genetic mutations but also from epigenetically driven oncogenic pathways [8]. As a result, epigenetic reprogramming has been recognized as a fundamental characteristic of cancer that contributes to the acquisition of various malignant traits [9].

Epigenetic dysregulation plays a central role in hepatocarcinogenesis. Among the various epigenetic mechanisms, DNA methylation is one of the most widely investigated and involves the methylation of cytosine residues within CpG dinucleotides, resulting in the formation of 5-methylcytosine. In LIHC, dysregulated DNA methylation manifests as both global hypomethylation, contributing to genomic instability, and focal hypermethylation of tumor suppressor gene promoters, leading to their transcriptional silencing [4,10]. Recent years have seen the identification of DNA methylation-based biomarkers for LIHC diagnosis and prognosis. Xu et al. demonstrated the efficacy of using circulating cell-free DNA methylation as a biomarker for early LIHC, providing its potential for high-precision screening through plasma analysis [11]. Building on this, Zhu et al. conducted a comprehensive analysis of methylated circulating tumor DNA, revealing panels of CpG sites with strong prognostic power and potential utility in monitoring treatment response [12].

NcRNAs constitute a functional group of transcripts, that, despite lacking protein-coding capacity, act as pivotal regulators of genomic activities, including gene expression, chromatin architecture, and cell signaling. Among these, microRNAs (miRNAs) are shorth RNA molecules of approximately 22 nucleotides in length, generated from hairpin-structured precursors. These miRNA function by guiding the RNA-induced silencing complex to complementary sites within target mRNAs, thereby inducing post-transcriptional repression. It has been estimated that miRNAs regulate a substantial proportion of human protein-coding genes, accounting for approximately 60%, and are critically involved in a wide biological process, including cellular development, differentiation, and the pathogenesis of diseases [13]. Especially, these molecules play significant roles in the initiation and progression of various cancers through their interaction with oncogenes and tumor suppressor genes [14,15]. Due to their distinct stability and characteristic expression profiles, miRNAs are increasingly recognized as promising biomarkers for predicting disease prognosis as well as potential targets for therapeutic intervention [16]. Furthermore, emerging evidence suggests that miRNA expression profiles may be useful for predicting the recurrence of LIHC following liver transplantation [17]. Long non-coding RNAs (lncRNAs), which are generally defined as transcripts exceeding 200 nucleotides in length, originated from various genomic contexts, including intergenic regions, antisense orientations, introns, and processed pseudogenes. These molecules participate in diverse mechanisms such as scaffolding of chromatin modifiers and modulation of RNA stability [18]. Although lncRNAs remain relatively less characterized compared to other transcript types, they are primarily synthesized by RNA polymerase II and have been increasingly recognized as contributors to cancer development, including LIHC [19]. Emerging research suggests that lncRNAs associated with LIHC are crucial regulators in the disease’s development and progression [20]. Furthermore, aberrant expression of lncRNAs has been linked to a variety of cellular processes, including proliferation, differentiation, apoptosis, invasion, and metastasis [21].

Pseudogenes, which number nearly 15,000 in the human genome, originate via retrotransposition or gene duplication events and were once considered non-functional genomic “fossils.” However, mounting evidence demonstrates that many pseudogenes produce stable ncRNAs with regulatory capacity in both normal physiology and disease [22]. Pseudogene-derived lncRNAs and ncRNAs can influence miRNA activity via the competing endogenous RNA (ceRNA) regulatory axis. This process is based on the presence of shared miRNA response elements, which enable these transcripts to bind and sequester miRNAs, thereby alleviating their inhibitory effects on target mRNAs [23,24]. The dysregulation of ceRNA networks has been implicated with carcinogenesis and cancer progression, as well as in the development of resistance to therapeutic interventions, suggesting their possible application in biomarker development and therapeutic strategies. Consistent with this notion, pseudogenes have emerged as players in tumor biology [25,26], exerting either oncogenic or tumor-suppressive roles across various malignancies, including LIHC [27]. Although several pseudogenes have been demonstrated to display aberrant expression patterns and association with the development of LIHC [28], only a limited number have been methodically investigated in the context of early-stage recurrence. Therefore, further studies are necessary to clarify the clinical significance and mechanistic functions of pseudogenes in the progression and recurrence of LIHC.

Here, we aimed to systematically characterize CBX2 expression in LIHC at both the transcriptional and protein levels, while also assessing its association with clinicopathological characteristics and prognostic significance. Furthermore, we explore the epigenetic landscape of CBX2, with a specific focus on DNA methylation, and we develop methylation-sensitive protein–protein interaction networks. We also investigate the regulatory circuits associated with CBX2 and ncRNAs, identifying critical miRNAs and their associated lncRNA or pseudogene that may influence CBX2 expression. Our integrative analysis suggests that CBX2 may have potential as a prognostic biomarker and provides insight into epigenetic and post-transcriptional regulatory mechanisms that may be considered in the development of therapeutic strategies.

## 2. Materials and Methods

### 2.1. Analysis of CBX2 Transcription in LIHC

To evaluate CBX2 mRNA expression in LIHC, several publicly available bioinformatics platforms were utilized, including TIMER2.0, Wanderer, and UALCAN. These resources integrate multi-omics datasets derived from TCGA, enabling comprehensive cancer-related analyses. TIMER2.0 (http://timer.cistrome.org/; 21 October 2025) was applied to examine the pan-cancer expression profile of CBX2, including its expression in LIHC [29,30,31]. Wanderer (http://maplab.cat/wanderer/; 21 October 2025) facilitated gene-specific expression exploration and allowed visualization of expression levels in both tumor and normal tissues [32]. UALCAN (http://ualcan.path.uab.edu/; 21 October 2025) provided subgroup analyses based on clinical parameters such as tumor stage, grade, and patient race/age, in addition to visualizing normalized expression of CBX2 [31].

### 2.2. Analysis of CBX2 Protein Expression in LIHC via Immunohistochemistry (IHC)

IHC data obtained from the Human Protein Atlas (https://www.proteinatlas.org; 21 October 2025), a comprehensive resource providing proteomic information across various cancer types, were used to validate the expression level of CBX2 in LIHC [30,33]. IHC images of normal liver and LIHC tissues were systematically evaluated to determine CBX2 staining intensity and its subcellular distribution. These evaluations were based on representative images and were not subjected to quantitative image analysis.

### 2.3. Prognostic Analysis of CBX2 in LIHC

The prognostic values of CBX2 expression in LIHC were evaluated through Kaplan–Meier (KM) using the KM Plotter platform (http://kmplot.com/analysis/; 21 October 2025) [29,34].

### 2.4. DNA Methylation and Prognostic Analysis of CBX2 in LIHC

CBX2 methylation was analyzed using publicly available bioinformatics tools, including OncoDB, UALCAN, MethSurv, and SMART database. OncoDB (https://oncodb.org/; 22 October 2025) was used to examine the association between CBX2 methylation and mRNA expression, integrating TCGA methylation data [35]. UALCAN provided insights into promoter methylation differences between tumor and normal tissues and across tumor stages [36]. MethSurv (https://biit.cs.ut.ee/methsurv/; 22 October 2025) enabled site-specific methylation analysis across CpG islands in the CBX2 promoter, with corresponding survival analysis based on beta values [37]. SMART (http://www.bioinfo-zs.com/smartapp/; accessed 22 October 2025) facilitated correlation analysis between CBX2 promoter methylation and gene expression, and survival stratification based on methylation profiles [38]. Correlation analysis was interpreted based on statistical significance (*p* < 0.05), and the results were considered exploratory due to the observational nature of the datasets.

### 2.5. Construction of the ncRNA Regulatory Network and Prognostic Analysis of CBX2 in LIHC

Candidate miRNAs targeting CBX2 were identified using the miRNet (https://www.mirnet.ca; accessed 22 October 2025) database [39], which integrates data from multiple miRNA–target interaction resources. The platform integrates data from multiple miRNA–target databases to provide interaction networks. Predicted miRNA–target interactions were filtered based on database-supported evidence and correlation analysis, applying thresholds of statistical significance (*p* < 0.05) and correlation coefficients (|r| > 0.1), where applicable. To further investigate the regulatory network associated with CBX2-related miRNAs, the ENCORI database [40] was used. ENCORI (https://rnasysu.com/encori/; accessed on 23 October 2025) was used to identify miRNA–lncRNA and miRNA–pseudogene interactions, and to extract expression and prognostic data for each ncRNA in the LIHC. Only interactions supported by ENCORI CLIP-seq data or predicted interaction datasets were included, and subsequent analyses were restricted to statistically significant associations. It should be noted that all identified ncRNA interactions are based on bioinformatic predictions and correlation analyses, and no experimental validation was performed in this study. Therefore, the constructed network should be interpreted as a hypothesis-generating framework instead of a confirmed regulatory mechanism.

### 2.6. Cell Culture

HepG2 hepatocellular carcinomas were purchased from the Korean Cell Line Bank (Seoul, Republic of Korea) and cultured in MEM medium supplemented with 1% antimycotic/antibiotic solution and 10% fetal bovine serum (Gibco, Grans Island, NY, USA), under standard conditions (37 °C, 5% CO_2_).

### 2.7. siRNA Transfection

Control siRNA and CBX2 siRNA (Bioneer, Daejeon, Republic of Korea) were transfected into HepG2 cells using Lipofectamine RNAiMAX transfection reagent (Invitrogen^TM^, Thermo Fisher Scientific, Waltham, MA, USA) in accordance with the manufacturer’s protocol. Briefly, cells were seeded in 96-well or 6-well plates (Corning Inc., Corning, NY, USA) and allowed to grow until they reached approximately 80% confluence. Subsequently, 50 nM of siRNA duplexes were prepared with Opti-MEM Reduced Serum Medium (Gibco) and combined with an equal volume of diluted Lipofectamine RNAiMAX. The solution was left at room temperature for 10 min to allow siRNA–lipid complex formation, and then the mixture was gently added onto plated cells. Cells were then maintained at 37 °C in a humidified incubator with 5% CO_2_ for 24, 48 and 72 h.

### 2.8. Quantitative RT-PCR

Total RNA was extracted from control siRNA- or CBX2 siRNA-transfected HepG2 cells using QIAzol reagent (Qiagen, Germantown, MD, USA) in accordance with the manufacturer’s protocol. Then, cDNA was synthesized and quantitative real-time PCR was performed using the One-Step PrimeScript RT-PCR Kit (Perfect Real Time) (Takara Bio, Tokyo, Japan) with an ABI Prism 7000 Sequence Detection System (Applied Biosystems, Foster City, CA, USA). Relative transcription levels of CBX2 were determined using the 2^−^ΔΔCt method and normalized to GAPDH expression. The primer sequences used were as follows: CBX2 forward, 5′-GGAACATGAGAAGGAGGTGCAG-3′; CBX2 reverse, 5′-GAAGAGGAGGAACTGCTGGACT-3′; GAPDH forward, 5′-GAAATCCCATCACCATCTTCC-3′; GAPDH reverse, 5′-GAGGCTGTTGTCATACTTCTC-3′.

### 2.9. Cell Viability and IC_50_ Analysis

Cell viability was estimated using MTT assay. Briefly, HepG2 cells were transfected with control or CBX2 siRNA for 24 h, followed by treatment with varying concentrations of 1,3-bis(2-chloroethyl)-1-nitrosourea (BCNU), cisplatin, or etoposide for 48 h. Then, the media administrated anticancer drugs were removed and replaced with fresh media containing MTT reagent. Following incubation for 4 h at 37 °C under light-protected conditions, insoluble formazan crystals were generated. The supernatant was then removed, and dimethyl sulfoxide was added to each well to solubilize the crystals. The resulting solution was quantified by measuring absorbance at 570 nm using a Falcon™ 96-Well, Cell Culture-Treated, Flat-Bottom Microplate (Falcon^®^, Corning Inc.). IC_50_ values were calculated by nonlinear regression analysis to determine cytotoxicity.

### 2.10. Wound-Healing Assay

HepG2 cells transfected with control or CBX2 siRNA were grown in 6-well plates to approximately 90% confluence. A straight-line scratch was made across the monolayer using a sterile 200 µL pipette tip to create a cell-free gap. Cells were subsequently rinsed twice with phosphate-buffered saline to remove residual debris and detached cells, then cultured in serum-free medium. The progression of wound closure was observed and photographed at 0, 24, 48, and 72 h using a phase-contrast microscope. Wound area was measured using ImageJ software (version 1.54p; National Institutes of Health, Bethesda, MD, USA), and the rate of wound closure was calculated according to the following formula: Wound closure (%) = (Area at 0 h—Area at each time point)/Area at 0 h × 100.

### 2.11. Statistical Analysis

Gene expression profile and clinical outcome data were retrieved from TCGA and GTEx datasets, which are integrated into platforms such as TIMER2.0, UALCAN, and Wanderer. Survival analyses were performed using several online tools, such as KMplot, MethSurv, and ENCORI. Pearson’s correlation analysis was applied to assess co-expression relationships among variables. To control for multiple testing, false discovery rate (FDR) correction was implemented. Statistical significance was considered at *p* < 0.05 or FDR < 0.05, as appropriate. For in vitro experimental, statistical analysis was carried out using GraphPad Prism software (version 10.4.1; GraphPad Software, San Diego, CA, USA). Data were presented as mean ± SEM. Difference between groups was analyzed using two-way ANOVA followed by Tukey’s post hoc multiple comparisons test, with significance set at *p* < 0.05.

## 3. Results

### 3.1. Transcription of CBX2 in LIHC

**Figure 1:**
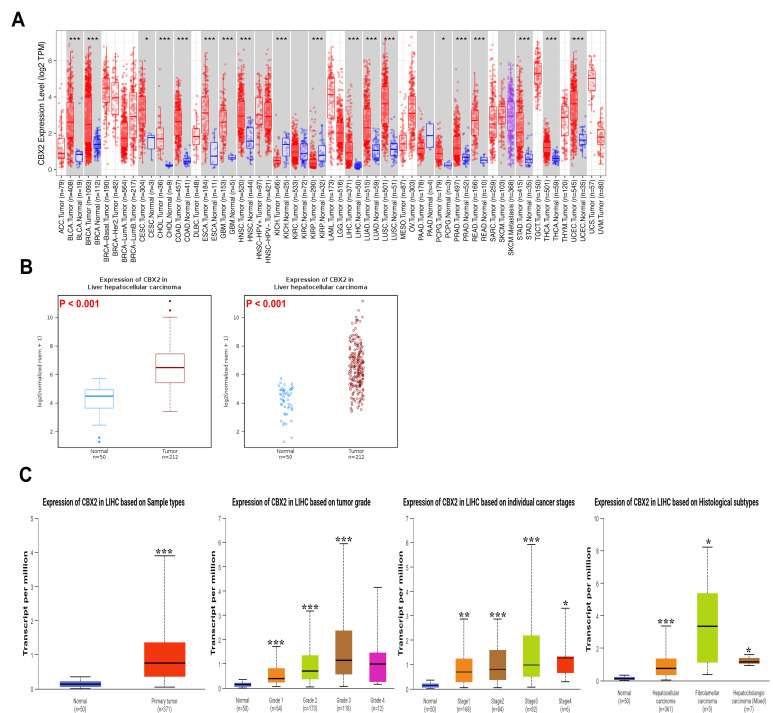
mRNA expression profile of CBX2 in LIHC. (**A**) Differential expression of CBX2 between tumor and normal tissues across multiple cancer types. (**B**) Relative CBX2 expression in LIHC compared with normal liver tissues. (**C**) CBX2 expression according to clinicopathological subgroups in comparison with normal samples. * *p* < 0.05, ** *p* < 0.01, *** *p* < 0.001.

CBX2 transcription was significantly elevated in multiple tumor types compared to corresponding normal tissues (Figure 1A). Notably, CBX2 was markedly upregulated in LIHC. Analysis using the Wanderer databases showed relatively higher expression levels of CBX2 in tumor tissue compared with normal liver tissue (Figure 1B). Comparison across sample types also indicated a tendency toward increased CBX2 expression in primary tumor tissues. When stratified by tumor grade, CBX2 expression showed a progressive increase from grade I to grade III tumors. Similarly, CBX2 expression levels varied significantly across cancer stages, with a gradual increase from stage I to stage IV. Subgroup analysis according to histological subtypes of LIHC indicated that CBX2 expression tended to be higher in hepatocellular carcinoma tissues compared with normal samples (Figure 1C). These observations suggest a potential increase in CBX2 expression in LIHC and imply a possible association with tumor characteristics and disease progression.

### 3.2. Protein Expression of CBX2 in LIHC

**Figure 2:**
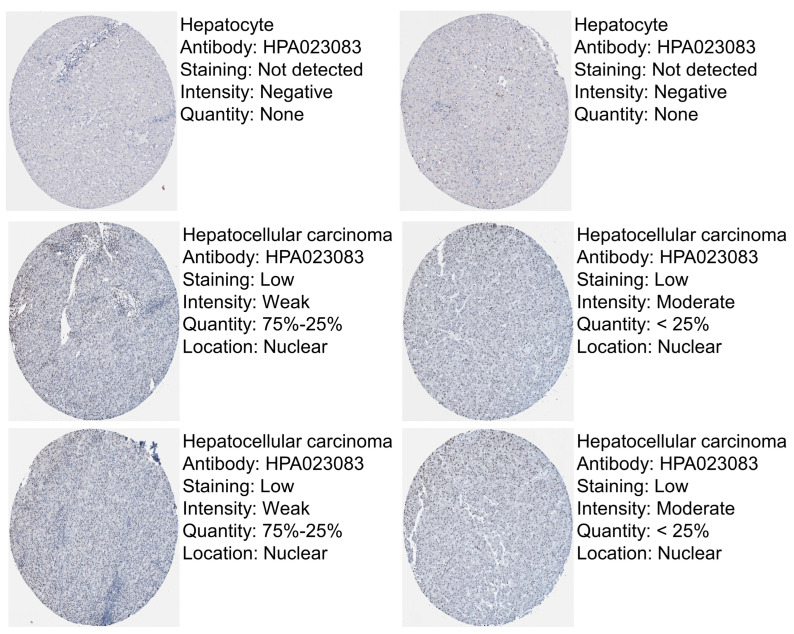
Immunohistochemical staining of CBX2 in normal liver and LIHC tissues. Representative images were retrieved from the Human Protein Atlas (www.proteinatlas.org, v25.0).

CBX2 protein expression in LIHC was analyzed using data obtained from the Human Protein Atlas database. The results showed that CBX2 protein expression was detectable in LIHC tumor tissues, whereas little to no expression was observed in normal hepatic tissues (Figure 2). However, as the analysis is based on representative images without quantitative evaluation, these findings should be interpreted with caution. Collectively, these observations suggest that CBX2 may be upregulated in LIHC at the protein level, consistent with its increased mRNA expression.

### 3.3. Prognostic Value of CBX2 Expression in LIHC

**Figure 3:**
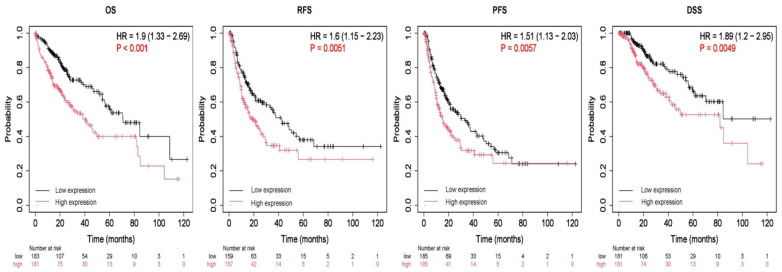
Prognostic relevance of CBX2 expression in LIHC. OS, overall survival; RFS, relapse-free survival; PFS, progression-free survival; DSS, disease-free survival.

The prognostic value of CBX2 expression in LIHC was evaluated by KM survival analysis using the KM database. Overall survival (OS), relapse-free survival (RFS), progression-free survival (PFS), and disease-free survival (DSS) were assessed as clinical endpoints. Increased CBX2 expression was significantly associated with worse prognosis, including shorter OS (hazard ratio (HR) = 1.9, *p* < 0.001), RFS (HR = 1.60, *p* = 0.0051), PFS (HR = 1.51, *p* = 0.0057), and DSS (HR = 1.89, *p* = 0.0049) (Figure 3). These results imply that elevated CBX2 expression may be associated with unfavorable clinical outcomes in patients with LIHC.

### 3.4. Correlation of CBX2 Expression with DNA Methylation in LIHC

**Figure 4:**
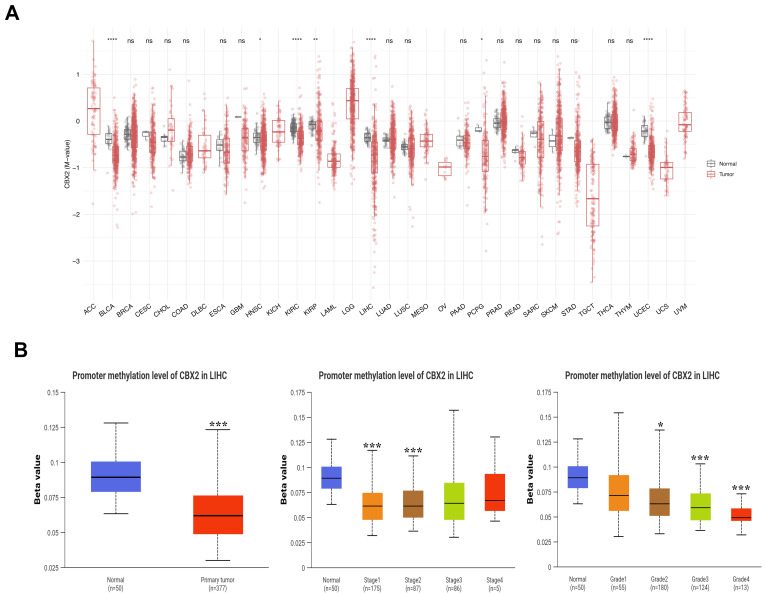
DNA methylation profiles of CBX2 in LIHC. (**A**) Differential methylation of CBX2 between tumor and normal across multiple cancer types. (**B**) Variation in CBX2 methylation levels according to clinicopathologic characteristics in LIHC and normal samples. * *p* < 0.05, ** *p* < 0.01, *** *p* < 0.001 and **** *p* < 0.0001.

To explore the possible contribution of DNA methylation to CBX2 expression in LIHC, an integrative analysis was applied using data from the SMART, OncoDB, and MEXPRESS databases. Comparative analysis of CBX2 promoter methylation across multiple cancer types revealed heterogenous methylation patterns, with LIHC exhibiting a distinct methylation profile (Figure 4A). In particular, promoter methylation of CBX2 was found to be decreased in LIHC tissues relative to normal tissues. Stratified analyses according to clinicopathological features revealed a progressive decrease in CBX2 promoter methylation with advancing tumor stage, with significantly lower methylation observed in stages I and II tumors relative to normal tissues. In addition, an inverse relationship was observed between CBX2 promotor methylation and tumor grade, with lower methylation levels detected in grade II, III, and Iv tumors relative to normal tissues (all *p* < 0.001) (Figure 4B). These results suggest that CBX2 promoter hypomethylation is a consistent epigenetic feature of LIHC and becomes more pronounced with tumor progression.

**Figure 5:**
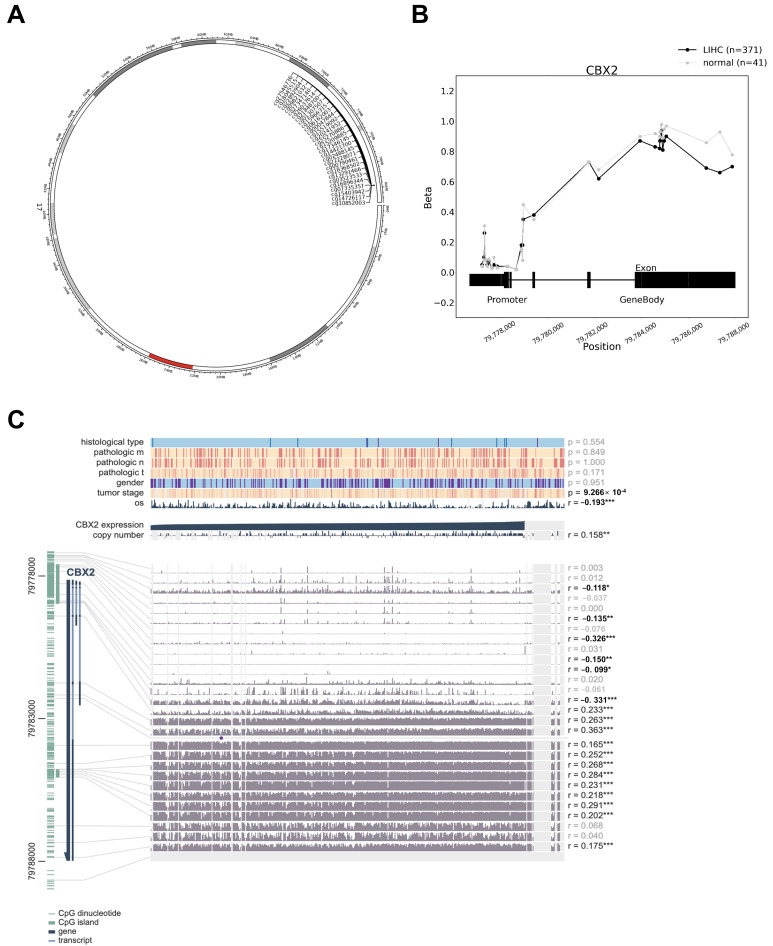
DNA methylation and its association with CBX2 expression in LIHC. (**A**) Distribution of DNA methylation probes across the CBX2 gene in LIHC. (**B**) Difference in CBX2 methylation between LIHC and normal liver tissues. (**C**) Relationship between CBX2 methylation status and gene expression in LIHC. * *p* < 0.05, ** *p* < 0.01, *** *p* < 0.001.

The chromosomal mapping of CBX2 revealed its localization on chromosome 17q25.3, as visualized in the circular ideogram (Figure 5A). Methylation profiling across the CBX2 promoter and gene body regions revealed distinct methylation patterns between LIHC and normal tissues (Figure 5B), with lower beta values at multiple promoter-associated CpG sites in LIHC. Comprehensive analysis of clinical variables, DNA methylation, and CBX2 expression demonstrated that CBX2 expression was significantly associated with tumor stage (*p* < 0.001), OS (r = 0.193, *p* < 0.001), and copy number variations (CNV; r = 0.158, *p* < 0.01). At the CpG site level, several CpG sites located within the promoter and first exon regions showed positive correlations with CBX2 expression, including cg15209885 (r = 0.233, *p* < 0.001), cg17346145 (r = 0.263, *p* < 0.001), cg14421700 (r = 0.363, *p* < 0.001), cg02488145 (r = 0.165, *p* < 0.001), cg22228071 (r = 0.252, *p* < 0.001), cg00790461 (r = 0.268, *p* < 0.001), cg18368502 (r = 0.284, *p* < 0.001), cg13291466 (r = 0.231, *p* < 0.001), cg03523533 (r = 0.218, *p* < 0.001), cg16896344 (r = 0.291, *p* < 0.001), cg07335357 (r = 0.202, *p* < 0.001), and cg10852003 (r = 0.175, *p* < 0.001). Conversely, several CpG sites exhibited significant negative correlations with CBX2 expression, including cg22892904 (r = −0.118, *p* < 0.001), cg26037614 (r = −0.135, *p* < 0.001), cg01307507 (r = −0.362, *p* < 0.001), cg06162003 (r = −0.150, *p* < 0.01), and cg00047844 (r = −0.099, *p* < 0.05) (Figure 5C). These results indicate a CpG site-specific relationship between DNA methylation and CBX2 expression.

**Figure 6:**
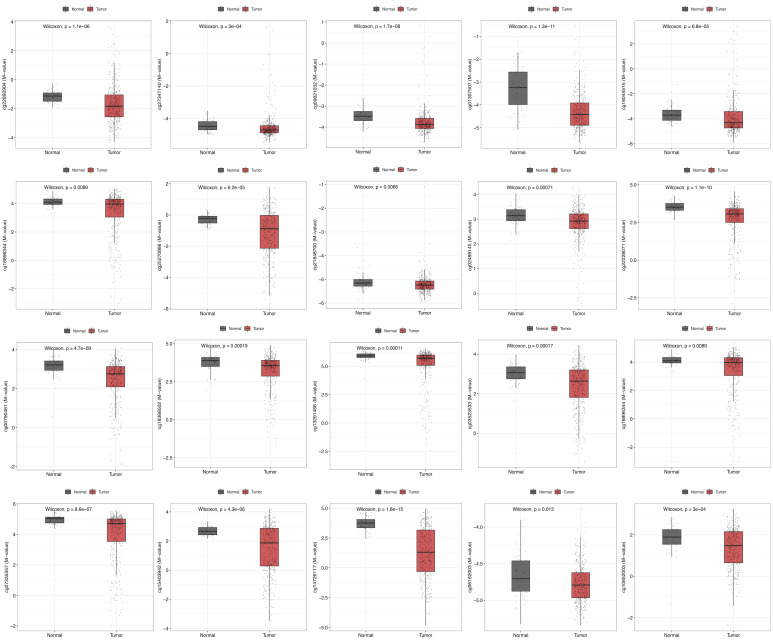
Comparison of CBX2-associated DNA methylation probes between normal and tumor.

To evaluate epigenetic dysregulation in LIHC, we systematically compared the DNA methylation levels of multiple CBX2-associated CpG sites between normal liver tissues and tumor tissues. A total of 20 specific CpG probes were analyzed. The results revealed that several CpG sites were significantly hypomethylated in LIHC, including cg22892904 (*p* < 0.001), cg27347140 (*p* < 0.001), cg09821032 (*p* < 0.001), cg01307507 (*p* < 0.001), cg18045515 (*p* < 0.001), cg16896344 (*p* = 0.0089), cg25270886 (*p* < 0.001), cg21848700 (*p* = 0.0066), cg02488145 (*p* < 0.001), cg22228071 (*p* < 0.001), cg00790461 (*p* < 0.001), cg18368502 (*p* < 0.001), cg13291466 (*p* < 0.001), cg03523533 (*p* < 0.001), cg16896344 (*p* = 0.0089), cg07335357 (*p* < 0.001), cg15403942 (*p* < 0.001), cg14726117 (*p* < 0.001), cg06162003 (*p* = 0.013), and cg10852003 (*p* < 0.001) (Figure 6).

**Figure 7:**
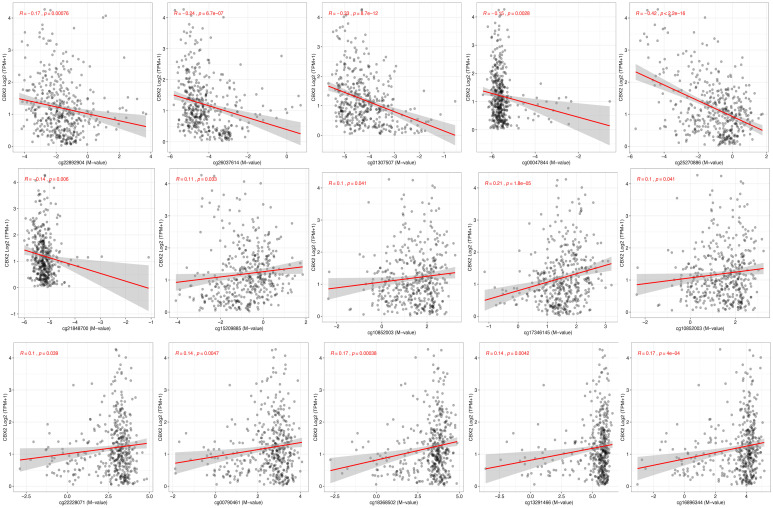
Correlation of CBX2-associated DNA methylation probes.

To examine how DNA methylation may influence CBX2 expression, correlation analyses were performed between CBX2 mRNA expression and methylation status at individual CpG sites. The results showed the presence of both inverse and positive associations. Significant negative correlations between methylation and CBX2 expression were observed at cg22892904 (r = −0.17, *p* < 0.001), cg26037614 (r = −0.24, *p* < 0.001), cg01307507 (r = −0.33, *p* < 0.001), cg00047844 (r = −0.15, *p* = 0.0028), cg25270886 (r = −0.42, *p* < 0.001), and cg21848700 (r = −0.14, *p* = 0.006). In contrast, significant positive correlations were identified at cg15209885 (r = 0.11, *p* = 0.033), cg10852003 (r = 0.10, *p* = 0.041), cg17346145 (r = 0.21, *p* < 0.001), cg22228071 (r = 0.10, *p* = 0.039), cg00790461 (r = 0.14, *p* = 0.0047), cg18368502 (r = 0.17, *p* < 0.001), cg13291466 (r = 0.14, *p* = 0.0042), and cg16896344 (r = 0.17, *p* < 0.001) (Figure 7). These results demonstrate that DNA methylation at distinct CpG sites is differentially associated with CBX2 gene expression in LIHC.

**Figure 8:**
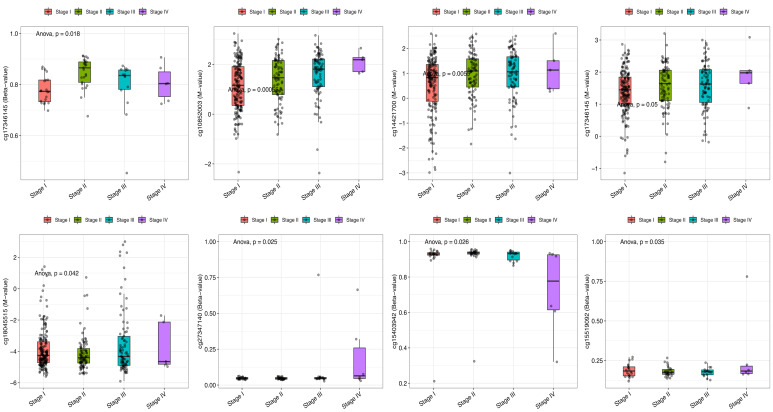
Pathological stages of CBX2-associatied DNA methylation probes in LIHC.

To explore the association between tumor stage and DNA methylation, methylation status at multiple CBX2-associated CpG sites were analyzed across clinical stages. Statistically significant differences in methylation levels were observed across LIHC stages, indicating stage-dependent epigenetic modulation. Stage-specific associations between DNA methylation and CBX2 expression were identified for cg17346145 (*p* = 0.018), cg10852003 (*p* < 0.001), cg14421700 (*p* = 0.0055), cg18045515 (*p* = 0.042), cg27347140 (*p* = 0.025), cg15403942 (*p* = 0.026), and cg15519092 (*p* = 0.035) (Figure 8). These findings suggest that stage-dependent DNA methylation changes at CBX2-associated CpG sites may contribute to LIHC progression.

**Figure 9:**
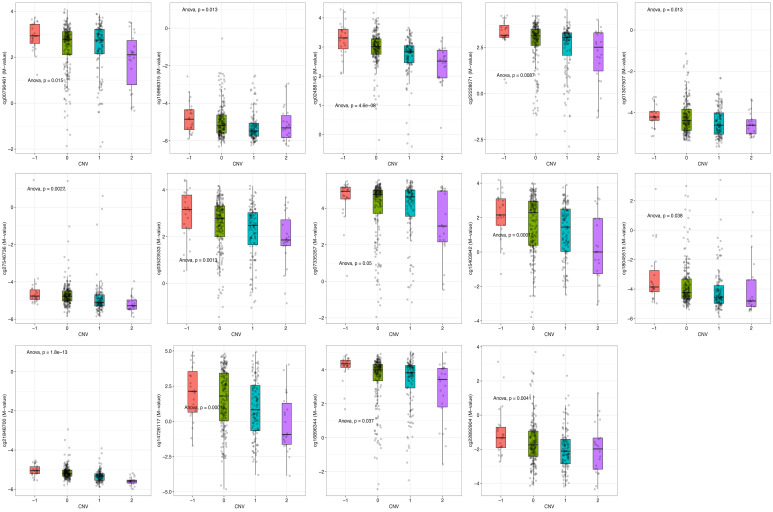
CNV of CBX2-associatied DNA methylation probes in LIHC. Colors indicate different pathological stages: Stage I (red), Stage II (green), Stage III (cyan), and Stage IV (purple).

To explore the relationship between CNV and DNA methylation at CBX2-associated CpG sites in LIHC, integrative analyses were performed. CNV status was significantly associated with methylation status at multiple CpG sites, such as cg00790461 (*p* = 0.015), cg15966315 (*p* = 0.013), cg02488145 (*p* < 0.001), cg22228071 (*p* = 0.0087), cg01307507 (*p* = 0.013), cg27546736 (*p* = 0.0027), cg03523533 (*p* = 0.0013), cg07335357 (*p* = 0.050), cg15403942 (*p* < 0.001), cg18045515 (*p* = 0.038), cg21848700 (*p* < 0.001), cg14726117 (*p* < 0.001), cg16896344 (*p* = 0.037), and cg22892904 (*p* = 0.0041) (Figure 9). These results indicate that CNV status is significantly associated with methylation levels at multiple CBX2-related CpG sites.

**Figure 10:**
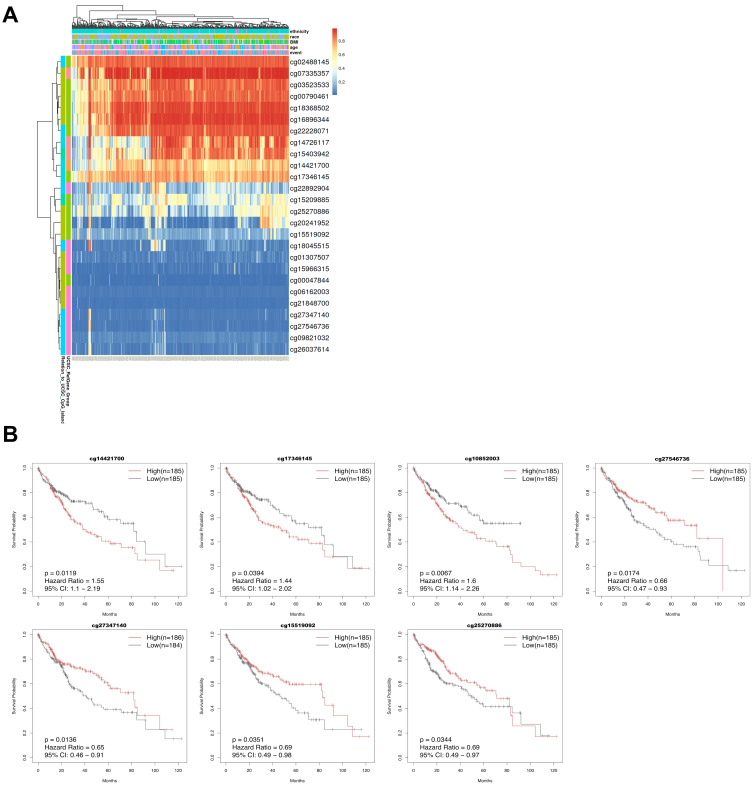
Prognostic significance of CBX2-associatied DNA methylation probes in LICH. (**A**) Heatmap illustrating CBX2 DNA methylation patterns. (**B**) KM survival curves based on CBX2 methylation status.

To characterize the global methylation landscape of CBX2-associated CpG sites in LIHC, hierarchical clustering analysis was performed. The resulting heatmap revealed distinct methylation patterns, including a cluster of hypermethylated probes (cg02488145, cg07335357, cg03523533, cg00790461, cg18368502, cg16896344, and cg22228071) and a cluster of hypomethylated probes (cg18045515, cg01307507, cg15966315, cg00047844, cg06162003, cg21848700, cg27347140, cg27546736, cg09821032, and cg26037614) across the LIHC cohort (Figure 10A). To evaluate the prognostic significance of CBX2-associated DNA methylation, Kaplan–Meier survival analyses were performed. High methylation levels at cg14421700 (HR = 1.55, *p* = 0.012), cg17346145 (HR = 1.44, *p* = 0.039), and cg10852003 (HR = 1.60, *p* = 0.007) were linked to worse prognosis, and low methylation levels at cg27347140 (HR = 0.65, *p* = 0.014), cg15519092 (HR = 0.69, *p* = 0.035), and cg25270886 (HR = 0.69, *p* = 0.034) were also associated with unfavorable outcomes (Figure 10B). Furthermore, univariate Cox proportional hazards regression analysis of 27 CpG probes identified six probes with significant prognostic value (Table 1), suggesting that specific CBX2-associated methylation signatures may serve as potential epigenetic biomarkers for poor prognosis in LIHC.

**Figure 11:**
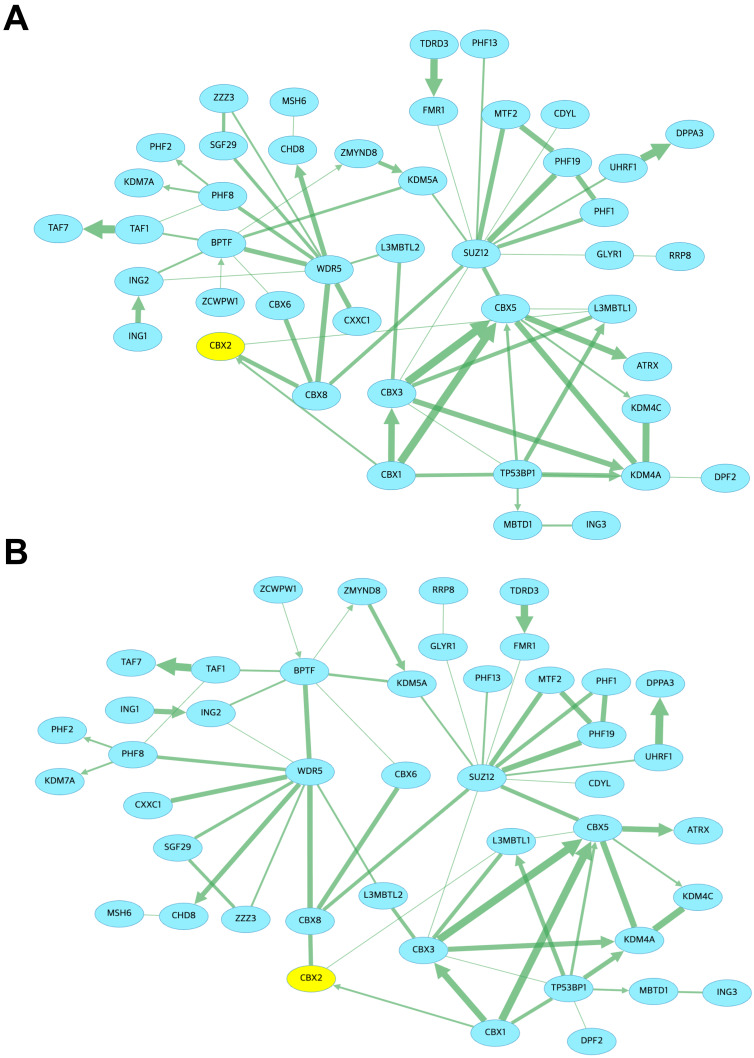
Protein–protein interaction networks of CBX2 in LIHC. (**A**) Methylation-dependent protein binding network of CBX2. (**B**) Methylated histone-binding network of CBX2.

To explore potential protein interactions influenced by methylation changes at CBX2 in LIHC, a methylation-sensitive protein–protein interaction network was constructed. In this network, CBX2 was functionally linked to other chromobox family members, including CBX1 and CBX8, suggesting coordinated activity within Polycomb Repressive Complex 1 (PRC1)-associated chromatin-silencing pathways (Figure 11A). Similarly, in the CBX2-associated methylated histone-binding network, CBX2 served as a central hub interacting with CBX1 and CBX8 (Figure 11B). These observations imply that CBX2 may act as an epigenetically modulated scaffold protein integrating DNA methylation signals into chromatin-based regulatory pathways in LIHC.

### 3.5. Prediction of CBX2-Associated miRNAs in LIHC

**Figure 12:**
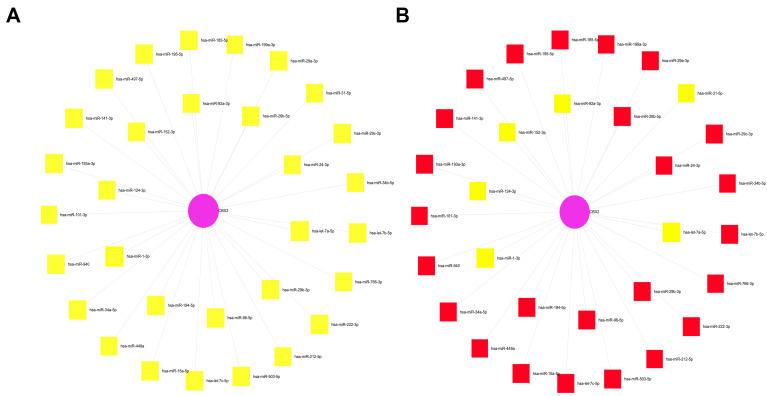
Predicted miRNAs targeting CBX2. (**A**) miRNA interaction network associated with CBX2. The magenta circle indicates CBX2, and the yellow squares represent miRNAs associated with CBX2. (**B**) CBX2-associated miRNA network in LIHC. The magenta circle indicates CBX2, while the yellow and red squares represent different groups of miRNAs associated with CBX2 in LIHC.

To identify potential miRNAs regulating CBX2 expression in LIHC, a miRNA–target interaction network was constructed. This network identified 30 miRNAs predicted to target CBX2, with CBX2 positioned as the central node and individual miRNAs represented as peripheral nodes (Figure 12A and Table 2). Among these, miRNAs previously implicated in LIHC were highlighted within the network (Figure 12B). These results suggest that CBX2 may be subject to miRNA-mediated post-transcriptional regulation involving both tumor-suppressive and oncogenic miRNAs, underscoring potential non-coding RNA interactions associated with CBX2 dysregulation in LIHC.

**Table 2:** miRNA associated with CBX2.

Gene	miRNA	
CBX2	hsa-let-7a-5p	hsa-miR-1-3p
	hsa-let-7b-5p	hsa-miR-124-3p
	hsa-let-7c-5p	hsa-miR-141-3p
	hsa-miR-15a-5p	hsa-miR-152-3p
	hsa-miR-24-3p	hsa-miR-185-5p
	hsa-miR-26b-5p	hsa-miR-193a-3p
	hsa-miR-29a-3p	hsa-miR-194-5p
	hsa-miR-31-5p	hsa-miR-195-5p
	hsa-miR-92a-3p	hsa-miR-29c-3p
	hsa-miR-96-5p	hsa-miR-34b-5p
	hsa-miR-101-3p	hsa-miR-449a
	hsa-miR-29b-3p	hsa-miR-497-5p
	hsa-miR-199a-3p	hsa-miR-503-5p
	hsa-miR-34a-5p	hsa-miR-766-3p
	hsa-miR-222-3p	hsa-miR-940
	hsa-miR-212-5p	

### 3.6. Expression, Prognostic Value, and Correlation of CBX2-Associated miRNAs in LIHC

**Figure 13:**
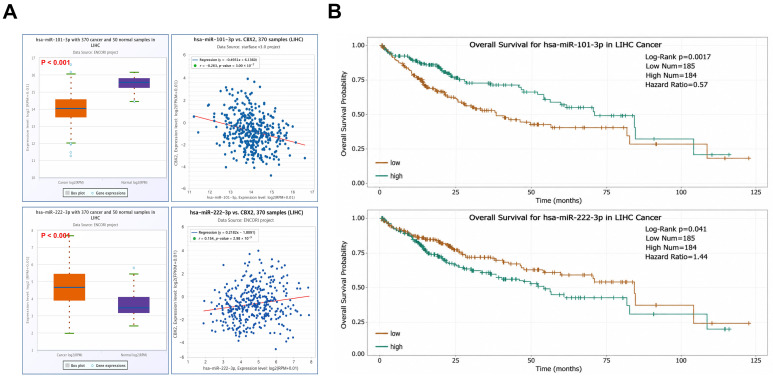
Expression patterns and prognostic relevance of CBX2-related miRNAs in LIHC. (**A**) Differential expression levels and correlation analyses of hsa-miR-101-3p and hsa-miR-222-3p with CBX2 in LIHC. (**B**) KM survival curves for hsa-miR-101-3p and hsa-miR-222-3p in LIHC.

Among the predicted miRNAs linked to CBX2 in LIHC, hsa-miR-101-3p and hsa-miR-222-3p were found to be associated with CBX2 expression. Therefore, to assess the functional impact of these miRNAs (hsa-miR-101-3p and hsa-miR-222-3p), their expression levels, correlation with CBX2 mRNA expression, and prognostic significance were systemically analyzed. Expression analysis indicated that hsa-miR-101-3p levels were reduced in LIHC relative to normal tissues (*p* < 0.001), whereas hsa-miR-222-3p levels were increased (*p* < 0.001). Correlation analysis showed that hsa-miR-101-3p expression was inversely related to CBX2 mRNA levels (r = −0.263, *p* < 0.001), while hsa-miR-222-3p exhibited a positive relationship with CBX2 expression (r = 0.154, *p* = 0.0030) (Figure 13A). Survival analysis using KM curves suggested that lower expression of hsa-miR-101-3p may be associated with less favorable clinical outcomes in patients with LIHC (HR = 0.57, *p* = 0.0017). Conversely, elevated hsa-miR-222-3p expression appeared to be associated with worse prognosis in LIHC (HR = 1.44, *p* = 0.041) (Figure 13B). Taken together, these results indicate that hsa-miR-101-3p and has-miR-222-3p may be related to CBX2 expression and clinical outcome in LIHC. While has-miR-101-3p exhibits features consistent with a tumor-suppressive role and hsa-miR-222-3p with an oncogenic role, these observations are based on correlated analysis and do not establish a direct regulatory relationship. These results describe potential miRNA-mediated regulatory interactions associated with CBX2 expression in LIHC.

### 3.7. Construction of CBX2-Associated ncRNA Regulatory Networks in LIHC

To characterize the ncRNA regulatory landscape linked with CBX2 in LIHC, we constructed two miRNA-centered ncRNA interaction networks for hsa-miR-101-3p and hsa-miR-222-3p. The networks include lncRNAs (Pink), circRNAs (Yellow), pseudogenes (Red), and sncRNAs (Green), which were predicted to interact with the respective miRNAs.

**Figure 14:**
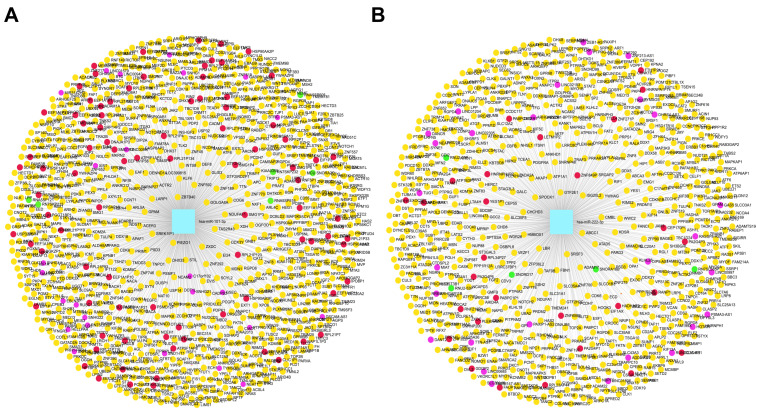
ncRNAs networks of CBX2-related miRNAs. (**A**) ncRNAs networks of hsa-miR-101-3p. (**B**) ncRNAs networks of hsa-miR-101-3p. Pink, lncRNAs; yellow, circRNAs; red, pseudogenes; green, sncRNAs.

The hsa-miR-101-3p-centered network includes a highly complex structure enriched with numerous lncRNAs, such as NEAT1, LINC00472, MALAT1, and HOTAIRM1, which are known to modulate gene expression and tumor behavior in LIHC. In addition, a large number of circRNAs, including circRNA_0000520 and circRNA_100338, were identified, suggesting extensive miRNA-associated post-transcriptional regulatory potential. Pseudogenes such as PTENP1 and HMGA1P6 were also incorporated into the network, suggesting their potential to act as decoys for miR-101-3p and regulate CBX2 expression. A few sncRNAs were present, further contributing to the overall complexity of the RNA-RNA interaction network (Figure 14A). Similarly, the hsa-miR-222-3p-centered network displayed a broad and intricate architecture. This network was enriched in lncRNAs such as TUG1, LINC00963, and SNHG3, alongside multiple circRNAs predicted to interact with has-miR-222-3p. In addition, pseudogenes including PPIAP43 and RPL23AP7 were also identified as targets, suggesting their involvement in competitive miRNA binding. Several sncRNAs were also found in the network, although they exhibited relatively fewer connections within the network (Figure 14B). Collectively, these results describe that hsa-miR-101-3p and hsa-miR-222-3p are embedded in complex and multilayered ncRNA interaction networks composed of diverse RNA species. The identified lncRNAs, cirRNAs, and pseudogenes may represent potential interacting partners of these miRNAs. However, the current analysis is based on predicted interactions and expression correlations, and does not provide sufficient evidence to support a functional ceRNA regulatory mechanism of CBX2.

### 3.8. Expression, Correlation, and Prognostic Value Analysis of CBX2-Associated lncRNAs and Pseudogenes in LIHC

**Figure 15:**
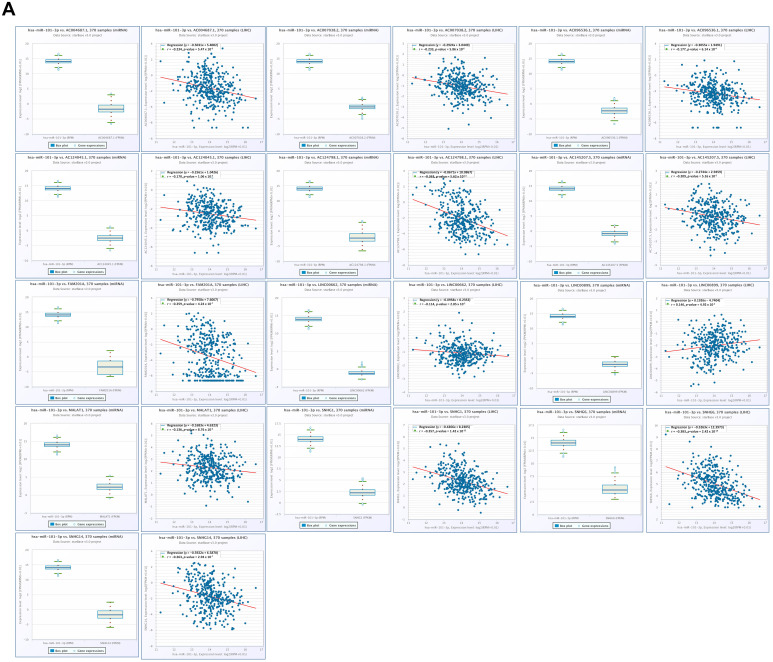

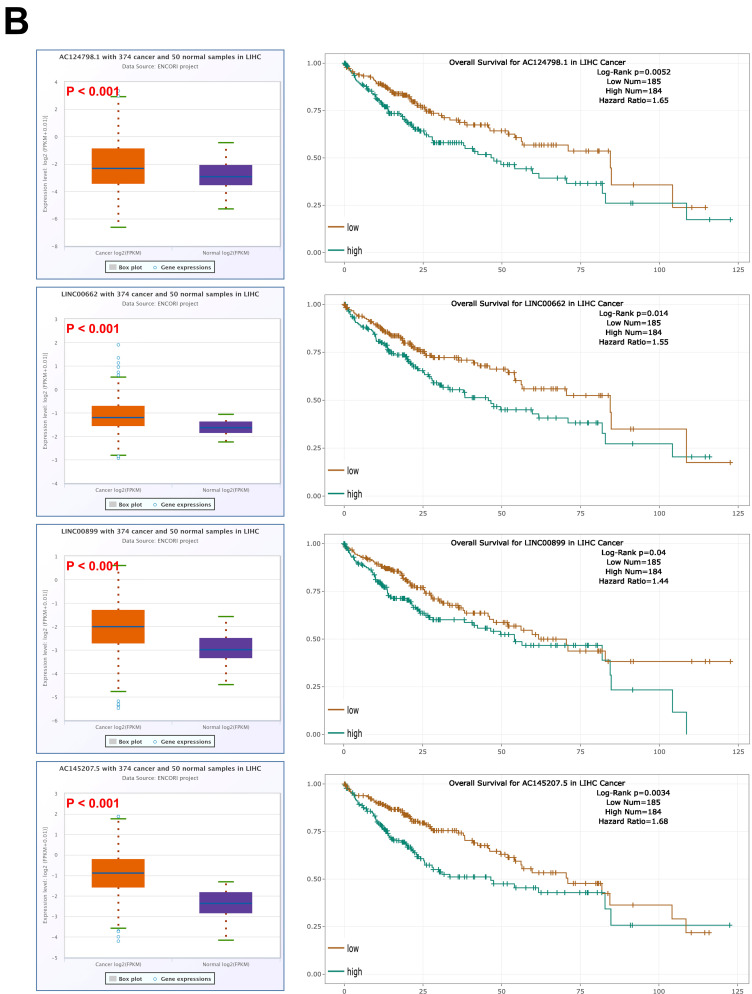
lncRNAs associated with hsa-miR-101-3p in LIHC. (**A**) Correlation of hsa-miR-101-3p-associated IncRNAs in LIHC. (**B**) Prognostic value of hsa-miR-101-3p-associated IncRNAs in LIHC (left), and KM plot of hsa-miR-101-3p-associated lncRNAs (right).

To identify lncRNAs and pseudogenes that may function as ceRNAs regulating CBX2 through sequestration of hsa-miR-101-3p or hsa-miR-222-3p in LIHC, we systemically analyzed their expression profiles, correlation patterns, and prognosis relevance. Correlation analysis revealed that increased hsa-miR-101-3p was significantly and negatively correlated with multiple lncRNAs, including AC004687.1 (r = −0.234, *p* < 0.001), AC0070382.3 (r = −0.233, *p* < 0.001), AC96536.1 (r = −0.177, *p* < 0.001), AC124045.1 (r = −0.170, *p* < 0.001), AC124798.1 (r = −0.363, *p* < 0.001), AC145207.5 (r = −0.209, *p* < 0.001), FAM201A (r = −0.259, *p* < 0.001), LNC00662 (r = −0.114, *p* = 0.029), LNC00899 (r = −0.146, *p* = 0.00492), MALAT1 (r = −0.136, *p* < 0.0088), SNHG1 (r = −0.357, *p* < 0.001), SNHG6 (r = −0.383, *p* < 0.001), and SNHG14 (r = −263, *p* < 0.001) (Figure 15A). To further assess the clinical relevance of CBX2-associated lncRNAs, differential expression analysis and KM survival analysis were performed for four candidate lncRNAs, such as AC124798.1, LINC00662, LINC00899. AC124798.1, and AC145207.5. All four lncRNAs exhibited increased expression in LIHC compared with normal liver tissues (all *p* < 0.001). Survival analysis indicated that higher expression levels of AC124798.1 (HR = 1.65, *p* = 0.0052), LINC00662 (HR = 1.55, *p* = 0.014), LINC00899 (HR = 1.44, *p* = 0.040), and AC145207.5 (HR = 1.68, *p* = 0.0034) were associated with unfavorable OS in patients with LIHC (Figure 15B).

**Figure 16:**
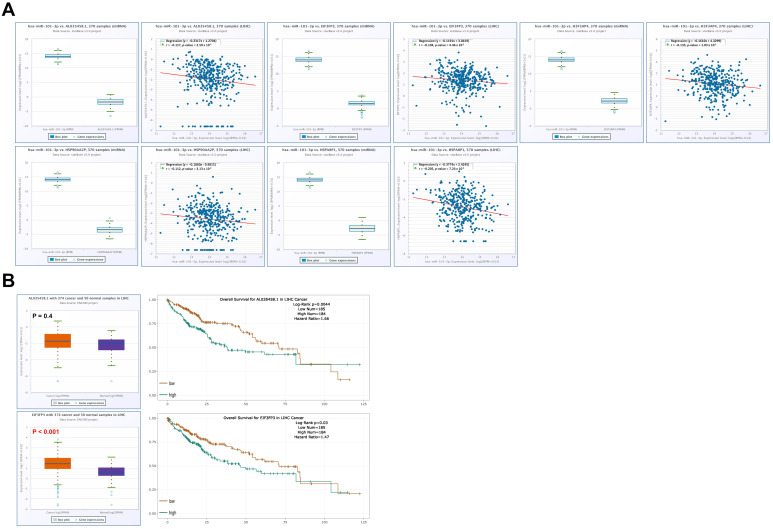
Pseudogenes associated with hsa-miR-101-3p in LIHC. (**A**) Correlation of hsa-miR-101-3p-associated pseudogenes in LIHC. (**B**) Prognostic value of hsa-miR-101-3p-associated pseudogenes in LIHC (left), and KM plot of hsa-miR-101-3p-associated pseudogenes (right).

In addition, hsa-miR-101-3p was negatively correlated with several pseudogenes, including AL035458.1 (r = −0.117, *p* = 0.03), EIF3FP3 (r = −0.104, *p* = 0.045), H3F3AP4 (r = −0.133, *p* = 0.0103), HSP90AA2P (r = −0.112, *p* < 0.0313), and HSPA8P1 (r = −0.205, *p* < 0.001) (Figure 16A). Prognostic analysis indicated that increased expression of AL035458.1 (HR = 1.66, *p* = 0.0044) and EIF3FP3 (HR = 1.47, *p* = 0.03) were associated with worse prognosis in LIHC (Figure 16B).

**Figure 17:**
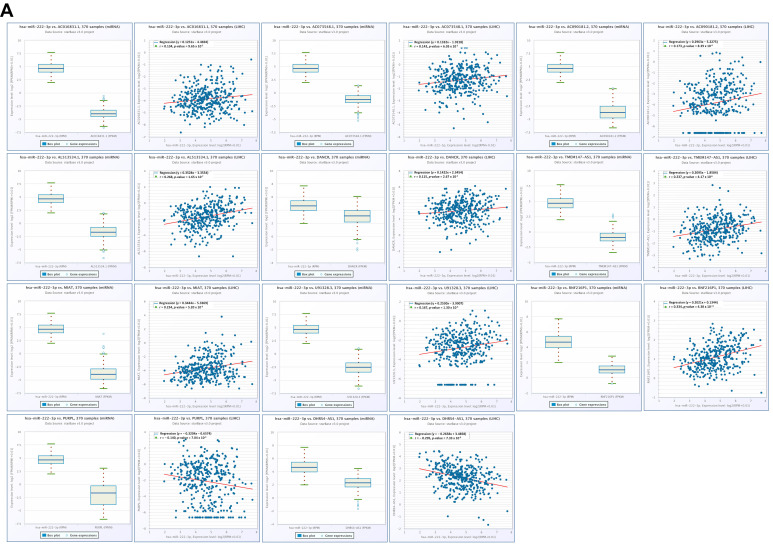

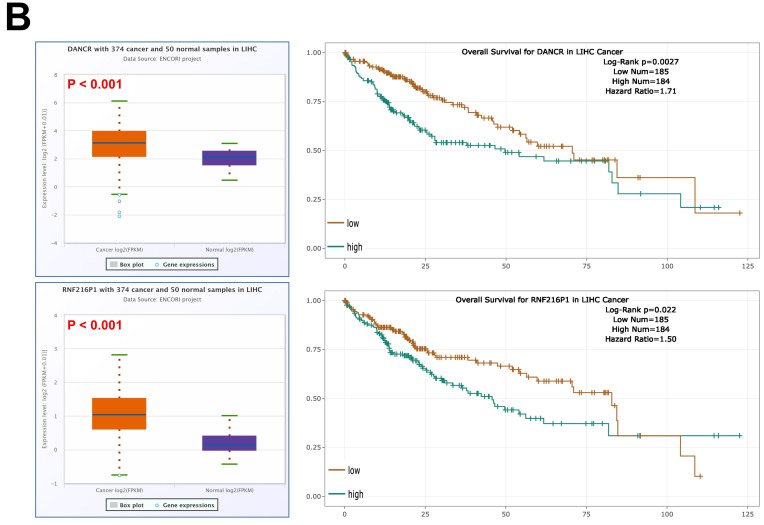
lncRNAs associated with hsa-miR-222-3p in LIHC. (**A**) Correlation of hsa-miR-222-3p-associated IncRNAs in LIHC. (**B**) Prognostic value of hsa-miR-222-3p-associated IncRNAs in LIHC (left), and KM plot of hsa-miR-222-3p-associated lncRNAs (right).

In contrast, hsa-miR-222-3p expression showed positive correlations with multiple lncRNAs, including AC016831.1(r = 0.134, *p* = 0.0097), AC073548.1 (r = 0.143, *p* = 0.00603), AC090181.2 (r = 0.173, *p* < 0.001), AL513534.1 (r = 0.268, *p* < 0.001), DANCR (r = 0.155, *p* = 0.027), TMEM147-AS1 (r = 0.237, *p* < 0.001), MIAT (r = 0.234, *p* < 0.001), U91328.3 (r = 0.167, *p* = 0.0013), and RNF216P1 (r = 0.334, *p* < 0.001). Negative associations were observed with PURPL (r = −0.140, *p* = 0.00704) and DHRS4-AS1(r = −0.295, *p* < 0.001) (Figure 17A). KM survival analysis indicated that increased expression of DANCR (HR = 1.71, *p* = 0.0027) and RNF216P1 (HR = 1.50, *p* = 0.022) was associated with less favorable clinical outcome in LIHC (Figure 17B).

**Figure 18:**
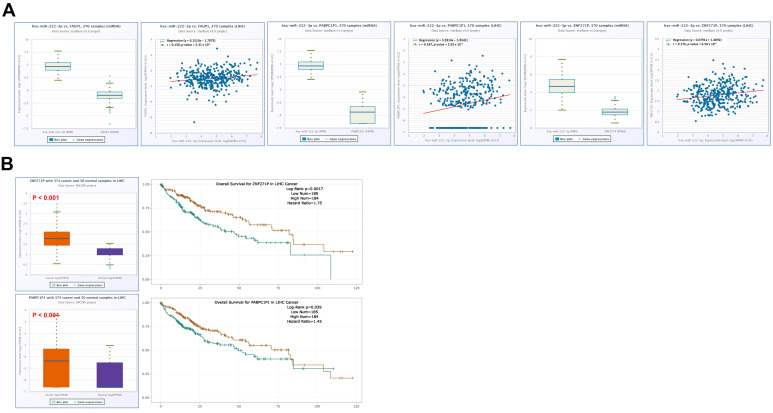
Pseudogenes associated with hsa-miR-222-3p in LIHC. (**A**) Correlation analysis of pseudogenes linked to hsa-miR-222-3p in LIHC. (**B**) Survival relevance of hsa-miR-222-3p-associated pseudogenes in LIHC (left), and KM plot of hsa-miR-222-3p-associated pseudogenes (right).

Furthermore, a significantly positive correlation between hsa-miR-222-3p expression and some pseudogenes, such as FAUP1 (r = 0.158, *p* = 0.00231), PABPC1P1 (r = 0.187, *p* < 0.001), and ZNF271P (r = 0.176, *p* < 0.001), was observed by correlation analysis (Figure 18A). Prognostic analysis indicated that elevated expression of ZNF271P (HR = 1.75, *p* = 0.0017) and (HR = 1.45, *p* = 0.039) was associated with worse survival outcomes in LIHC (Figure 18B). Overall, these results imply that multiple CBX2-associated lncRNAs and pseudogenes are correlated with miRNA expression patterns and patient prognosis in LIHC. These ncRNAs may be involved in miRNA-mediated regulatory networks; however, their functional roles in regulating CBX2 expression through a ceRNA mechanism remain to be further validated.

### 3.9. Effect of CBX2 Silencing on Chemosensitivity and Cell Migration in HepG2 Cells

**Figure 19:**
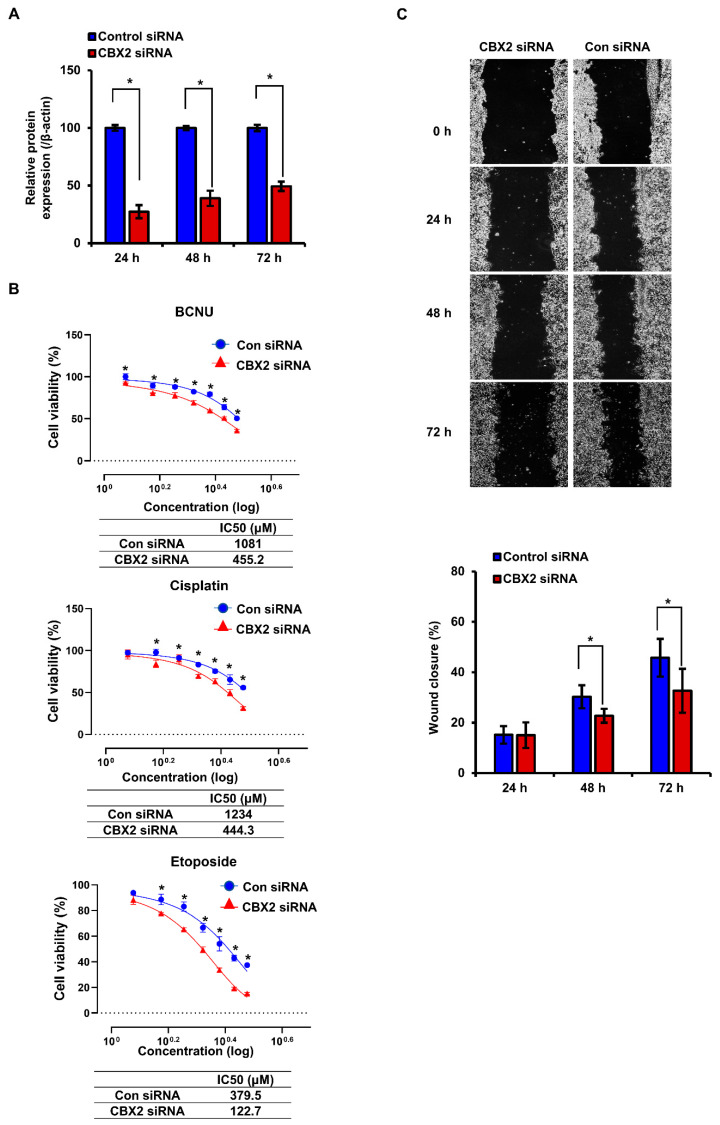
CBX2 knockdown enhances chemosensitivity and inhibits migration in HepG2 cells. (**A**) Quantitative RT-PCR analysis of *CBX2* mRNA expression in cells transfected with control siRNA or *CBX2* siRNA for 24, 48, and 72 h, respectively. *CBX2* mRNA levels were normalized to *GAPDH* and are presented relative to control siRNA. (**B**) Dose–response curves for BCNU, cisplatin, and etoposide in control siRNA- and CBX2-transfected cells. Cell viability was estimated by MTT assay at 48 h after drug treatment, and IC_50_ values are shown below each curve. (**C**) Representative wound-healing images at 0, 24, 48, and 72 h after scratching, and quantitative analysis of wound closure. Data are presented as mean ± SD. * *p* < 0.05 versus control siRNA.

To investigate the potential role of CBX2 in chemoresistance and cell migration, changes in cell viability following anticancer drug treatment and migratory capacity were evaluated in HepG2 cells after siCBX2 transfection. Western blotting showed the decrease in CBX2 protein expression in siCBX2-transfected cells, indicating CBX2 expression was effectively reduced after siCBX2 transfection (Figure 19A). Specifically, the reduction was most prominent at 24 h after transfection and gradually attenuated over time. In the MTT assay, IC_50_ values for BCNU, cisplatin, and etoposide were decreased in siCBX2-transfected cells (Figure 19B), suggesting a potential association between CBX2 expression and chemosensitivity in LIHC. In addition, wound-healing assay showed reduced wound closure in siCBX2-transfected cells, compared with control cells (Figure 19C), indicating decreased migratory capacity. Collectively, these results suggest that CBX2 may be linked to chemoresistance and cell migration in HepG2 cells. However, these findings are based on a single cell line and transient knockdown, and the wound-healing assay was not controlled for cell proliferation; therefore, further investigations are required to validate these observations.

## 4. Discussion

Recent studies have highlighted the significance of epigenetic modifications and the regulatory roles of non-coding RNAs (ncRNAs) in the process of hepatocarcinogenesis, presenting potential avenues for diagnostic and therapeutic interventions [41,42,43,44]. In the present investigation, we conducted a thorough examination of the role of CBX2, a polycomb group protein implicated in chromatin remodeling, within the context of LIHC. Our results indicate that CBX2 is elevated at the mRNA level in LIHC and shows detectable protein expression in tumor tissues, correlates with advanced tumor stages and unfavorable prognosis, and is associated with epigenetic features and ncRNA-related interactions without providing evidence of a direct regulatory mechanism. The overexpression of CBX2 in LIHC has been supported through various datasets, demonstrating a progressive increase in expression correlating with tumor grade and stage. Immunohistochemical analyses based on publicly available data further supported these observations at the protein level, although these findings are based on representative images without quantitative evaluation. Furthermore, in vitro experiment suggested that CBX2 expression levels may be associated with chemosensitivity against anticancer drugs and migratory activity in HepG2 cells. However, these results are preliminary and require further validation using additional models and functional assays. These observations suggest a potential involvement of CBX2 in the progression of LIHC. Survival analyses suggested that higher CBX2 expression may be correlated with unfavorable prognosis, highlighting its potential as a prognostic indicator.

Furthermore, our investigation indicated a reduction in CBX2 promoter-associated methylation status in LIHC tissues relative to normal tissues, with a gradual hypomethylation trend observed across increasing tumor stages and grades. Site-specific methylation profiling identified several CpG sites that exhibited both inverse and positive correlations with CBX2 expression, indicating a CpG site-specific and context-dependent relationship instead of a uniform promoter hypomethylation-driven mechanism. Therefore, the association between DNA methylation and CBX2 expression warrants careful interpretation. This finding aligns with earlier studies highlighting multifaceted involvement of DNA hypomethylation in oncogene regulation in LIHC [45,46]. To further clarify the mechanisms potentially associated with the dysregulation of CBX2, we developed a prediction-based ncRNA interaction network that identifies miRNAs, lncRNAs, and pseudogenes that may affect CBX2 expression. Within this regulator network, hsa-miR-101-3p and hsa-miR-222-3p were identified as putative modulators. hsa-miR-101-3p, which has been described as having tumor-suppressive properties, was reduced in LIHC and exhibited an inverse relationship with CBX2 expression. Conversely, hsa-miR-222-3p showed an increased and positive association with CBX2 level. Notably, lower expression of hsa-miR-101-3p and higher expression of hsa-miR-222-3p were linked to unfavorable clinical outcomes, suggesting their potential biological and clinical relevance. The analysis of the lncRNA and pseudogene network has suggested a potential interaction framework as opposed to a validated ceRNA regulatory axis. Within this framework, lncRNAs such as AC124798.1, LINC00662, and members of the SNHG family may be associated with miR-101-3p, potentially influencing CBX2 expression. Similar association patterns were observed within the miR-222-3p network, where lncRNAs such as DANCR and pseudogenes like ZNF271P showed a positive correlation with CBX2 expression and were linked to adverse prognostic outcomes. However, these findings are based on predicted interactions and correlation analyses without direct experimental validation. Taken together, these results highlight a potentially complex epigenetic and post-transcriptional regulatory landscape associated with CBX2 expression in LIHC.

Our findings suggest that CBX2, in conjunction with its related methylation sites and ncRNA regulators, may have potential as a biomarker for the diagnosis and prognosis of LIHC; however, its therapeutic relevance requires further functional validation. The methylation status at particular CpG sites exhibited potential prognostic value. Construction of a methylation-sensitive protein–protein interaction network revealed that CBX2 may interact with CBX1 and CBX8, suggesting a potential role within the PRC1 complex. This finding suggests potential functional relationships, rather than confirmed interactions, among members of the CBX family in the context of chromatin-mediated gene regulation in LIHC. These observations add to the current understanding of epigenetic regulation in liver cancer, although additional experimental validation is required. This research presents a comprehensive in silico analysis; however, it is essential to validate these findings through the use of patient-derived samples and additional functional assays to clarify the biological significance of CBX2 and its associated regulatory elements. Furthermore, confirmation in independent patient cohorts would be helpful to support the reliability of these findings. Future studies should investigate the potential clinical and biological implications of CBX2 and its regulatory network, including possible utility of epigenetic inhibitors or ncRNA mimics/inhibitors as investigational strategies. Furthermore, incorporation of multi-omics approaches, particularly single-cell epigenomic data, could offer additional insight into tumor heterogeneity and context-specific regulatory mechanisms specific to individual cell types.

## 5. Conclusions

In conclusion, this study suggests that CBX2 may represent a candidate prognostic marker in LIHC, with its expression potentially being associated with DNA hypomethylation and ncRNA-mediated post-transcriptional regulatory mechanisms. The integrative in silico methodology employed in this study provides preliminary and hypothesis-generating evidence, positioning CBX2 and its associated regulatory network as putative candidates worthy of further investigation for potential biomarker application in the context of LIHC.

## Figures and Tables

**Table 1:** Forest plot of univariate Cox regression analysis of DNA methylation probes of CBX2 in LIHC.

DATASET	HR (95% CI)	*P*-Value
cg27546736	**0.660 (0.470–0.930)**	**0.017**
cg18045515	0.998 (0.894–1.115)	0.977
cg22892904	1.002 (0.882–1.138)	0.973
cg09821032	0.959 (0.738–1.245)	0.752
cg27347140	**0.650 (0.460–0.910)**	**0.014**
cg26037614	0.930 (0.786–1.100)	0.396
cg21848700	0.730 (0.451–1.181)	0.200
cg01307507	0.904 (0.728–1.123)	0.364
cg15966315	0.923 (0.750–1.135)	0.445
cg06162003	1.022 (0.536–1.948)	0.948
cg00047844	0.911 (0.679–1.224)	0.538
cg15519092	**0.690 (0.490–0.980)**	**0.035**
cg20241952	0.954 (0.886–1.028)	0.216
cg25270886	**0.690 (0.490–0.970)**	**0.034**
cg15209885	0.982 (0.838–1.151)	0.823
cg17346145	**1.440 (1.020–2.020)**	**0.039**
cg14421700	**1.550 (1.100–2.190)**	**0.012**
cg02488145	1.077 (0.805–1.442)	0.617
cg22228071	1.069 (0.929–1.231)	0.350
cg00790461	1.146 (0.960–1.368)	0.131
cg18368502	1.068 (0.921–1.238)	0.384
cg13291466	1.022 (0.912–1.145)	0.709
cg03523533	1.044 (0.893–1.222)	0.586
cg16896344	1.082 (0.955–1.228)	0.217
cg07335357	1.028 (0.918–1.151)	0.632
cg15403942	1.059 (0.955–1.174)	0.277
cg14726117	1.032 (0.952–1.119)	0.446
cg10852003	**1.600 (1.140–2.260)**	**0.007**

**Green**: protective (HR < 1, *p* < 0.05); **red**: risk (HR > 1, *p* < 0.05); black: non-significant. HR, hazard ratio; CI, confidence.

## Data Availability

All data are available upon reasonable request from the corresponding author.

## Abbreviations

The following abbreviations are used in this manuscript:

BCUN	1,3-bis(2-chloroethyl)-1-nitrosourea
CBX2	Chromobox protein homolog 2
ceRNA	Competing endogenous RNA
DSS	Disease-free survival
LIHC	Liver hepatocellular carcinoma
lncRNA	Long non-coding RNA
ncRNA	Non-coding RNA
OS	Overall survival
PFS	Progression-free survival
RFS	Relapse-free survival
